# Etiologic factors in developing sacrococcygeal pilonidal sinus disease in males; A cased-control study

**DOI:** 10.1007/s00423-025-03907-1

**Published:** 2025-10-28

**Authors:** Jochem de Kort, A. Akke Pronk, Marijke R. van Dijk, Annemiek Maaskant, Menno R. Vriens, Niels Smakman, Edgar J. B. Furnee

**Affiliations:** 1 Department of Surgery, Diakonessenhuis Utrecht, P.O. Box 80250, 3508 TG Utrecht, the Netherlands; 2 Department of Urology, Radboud University Medical Centre, Nijmegen, the Netherlands; 3 Department of Pathology, University Medical Centre Utrecht, Utrecht, the Netherlands; 4 Animal Science Department Biomedical Primate Research Centre, Rijswijk, the Netherlands; 5 Department of surgery, University Medical Centre Utrecht, Utrecht, the Netherlands; 6 Department of Surgery, University Medical Centre Groningen, Groningen, the Netherlands

**Keywords:** Pilonidal sinus disease, Proctology, Benign surgery, Etiology

## Abstract

**Aim:**

Two main theories have been proposed for the development of sacrococcygeal pilonidal sinus disease (SPSD), but the exact etiology remains unclear. Better understanding of etiologic factors could improve insight into the disease and help prevent both primary and recurrent pilonidal sinus disease. This study aimed to identify etiologic factors in the development of pilonidal sinus disease.

**Method:**

For this case control study, potential etiologic factors such as family history of SPSD, BMI, working in a sitting position, and smoking were evaluated in 83 patients with primary SPSD and 83 controls. Additionally, anatomical factors, including the depth, width, and hair density of the natal cleft were compared. Lastly, microscopic hair analysis assessed hair characteristics such as thickness, pigmentation, breakage, hair cuticle irregularities and hair thickness irregularities.

**Results:**

Patients with SPSD were significantly more often smokers and had a family history of SPSD. They also had a significantly shallower and wider natal cleft, a greater number of hairs in the natal cleft, and these hairs were significantly thicker with more cuticle and thickness irregularities compared to the controls. However, after multivariable analysis, only working in a sitting position, a shallower natal cleft and more hairs at the natal cleft were independently associated with SPSD.

**Conclusion:**

A shallower natal cleft, more hair at the natal cleft and working in a sitting position were independent etiologic factors of SPSD. Therefore, keeping the natal cleft free of hair and avoiding prolonged sitting position should be recommended as preventive measures when counselling patients with SPSD.

## Introduction

Pilonidal sinus disease is a disorder most commonly located at the sacrococcygeal area. Despite sacrococcygeal pilonidal sinus disease (SPSD) is a very common disease with a high disease burden and a rising incidence [1], the exact etiology still remains unclear. In the literature, the etiology of SPSD is based on two different theories; the congenital and acquired theory. The first mentioned theory suggests that the infection arises from a congenital subcutaneous sinus, already present at birth. This theory is supported by familial clustering of pilonidal sinus disease, although hereditary factors have not yet been identified [2]. One of the acquired theories states that hair follicles at the natal cleft get irritated by microtrauma, rubbing and/or crushing, thus forming “pits”. After this pit formation, either the primary natal cleft hairs or hairs originating from other parts of the body enter these pits, penetrating the tissue beneath the skin causing inflammation and infection. Another acquired theory states that the etiology of SPSD has no correlation with pre-acquired pits, but is caused by sharp cut hair. In this theory, it has been claimed that the sharp edges of the cut (occipital) hair puncture the intact skin at the natal cleft, possibly enabled by mechanical aspects and cofactors such as the anatomy of the natal cleft, like width and depth [3, 4].

Based on the abovementioned theories, several etiological factors in the development of SPSD have been suggested, including anatomical variations of the natal cleft, lack of hygiene, seated profession, strength of occipital hair and different aspects of hair growth in the natal cleft such as the number of hairs and hair thickness. Although a higher incidence of SPSD within certain families has been confirmed in the literature as well as an association with higher Body Mass Index (BMI) and local trauma, some knowledge gaps of etiologic factors in the development of SPSD still remain present [4–6]. For example, why does the onset of SPSD appear to be shifting to a younger age, especially in patients with early onset of puberty [7, 8], and why is the incidence in women lower than in men? Knowledge on etiologic factors could help understanding the unclear etiology of SPSD better, and consequently helps preventing the development of recurrent and even primary SPSD.

The aim of this study was to identify etiologic factors in the development of SPSD by comparing a group of male patients with primary SPSD with a group of males without SPSD.

## Methods

### Study population

#### Cases

For this comparative cohort study, cases, i.e. patients with a confirmed primary SPSD, were identified from a randomised controlled trial (RCT) previously performed at the Diakonessenhuis Utrecht, the Netherlands, comparing phenolization and excision as treatment for primary SPSD [9]. Patients older than 18 years with a primary chronic SPSD were included in this trial. Recurrent SPSD was an exclusion criterion. Since SPSD is far less common in females and anatomical aspects at the natal cleft differ between sexes, female patients were excluded, and only male patients included in the RCT were analysed in the current study to ensure a more homogeneous group. Written informed consent was obtained from all participants.

#### Controls

The cases were compared to male individuals without a history of SPSD in a 1:1 ratio. The latter individuals were selected from the outpatient clinic at the Department of Surgery in the Diakonessenhuis Utrecht (NL) who presented with a benign condition other than SPSD. These males (controls) were matched with the cases based on age. History of SPSD was the only exclusion criterion for the control group. Informed consent was obtained from all individuals who agreed to participate in the control group.

The study received approval from the Medical Ethics Committee in Utrecht, the Netherlands.

#### Data collection of potential etiologic factors

Baseline characteristics and individual factors potentially related to the etiology of SPSD were prospectively obtained through questionnaires in both groups. These potential etiologic factors included family history of SPSD, BMI, working in sitting position and smoking. Also, SPSD-related symptoms at the natal cleft, including pain, fluid discharge, and itching, were assessed in the questionnaire of the controls to ensure that controls did not have an occult SPSD.

**Fig. 1:**
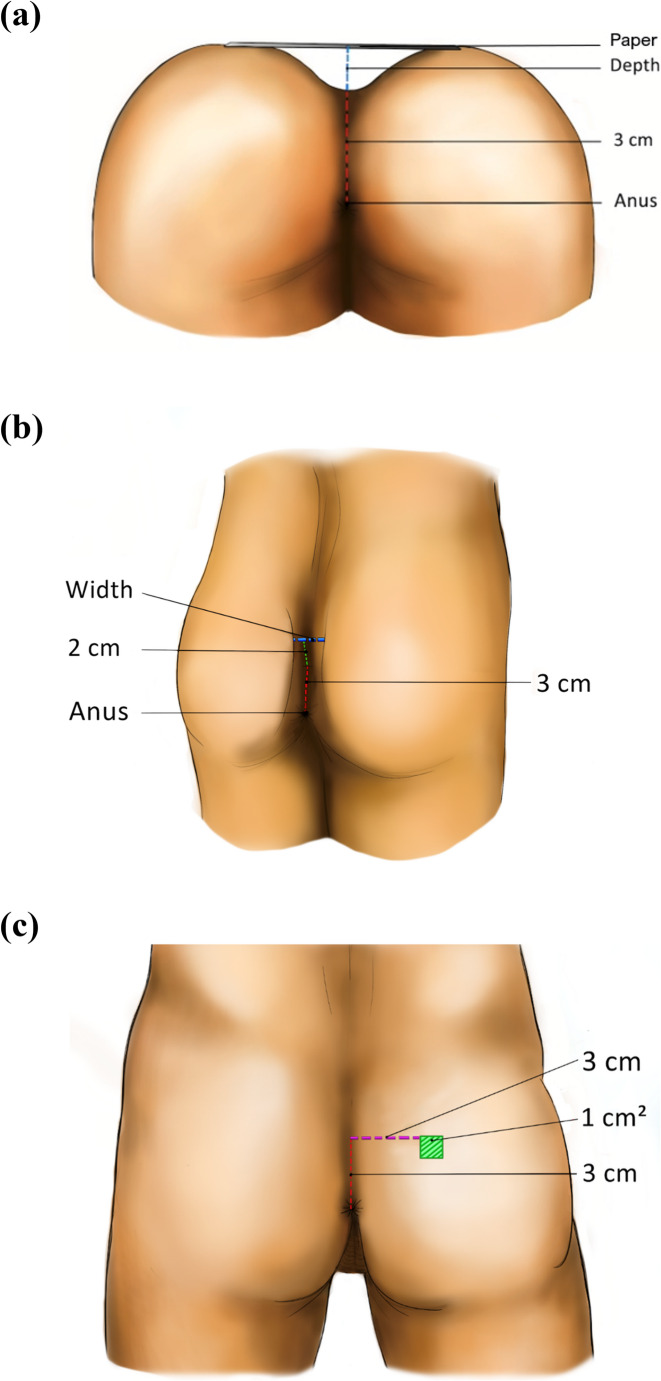
**A**. Schematic overview of the anatomical measurement at the depth at the sacrococcygeal area. **B**. Schematic overview of the anatomical measurement of the width at the sacrococcygeal area. **C**. Schematic overview of the anatomical measurements of the area that is shaved at the sacrococcygeal area

Anatomical factors potentially linked to the etiology of SPSD were intraoperatively assessed in the cases and in the outpatient clinic in the controls, including the depth and width of the natal cleft, and the number of hairs. Measurements were performed according to a predefined protocol to achieve identical measurement in all cases and controls (Figs. 1 A, B and C); the depth of the natal cleft was measured three centimeters superior to the dorsal edge of the anal verge, with a piece of paper (5 g) placed over the glutes as a reference for the height of the natal cleft, and the width of the natal cleft three centimeters superior to the dorsal edge of the anal verge and two centimeters above the midline. The number of hairs per square centimeter was assessed three centimeters superior to the dorsal edge of the anal verge and three centimeters lateral to the midline, on the left or right side. The measurements were performed in the case group by two authors (NS and EF), and in the control group by JK and NS.

**Fig. 2:**
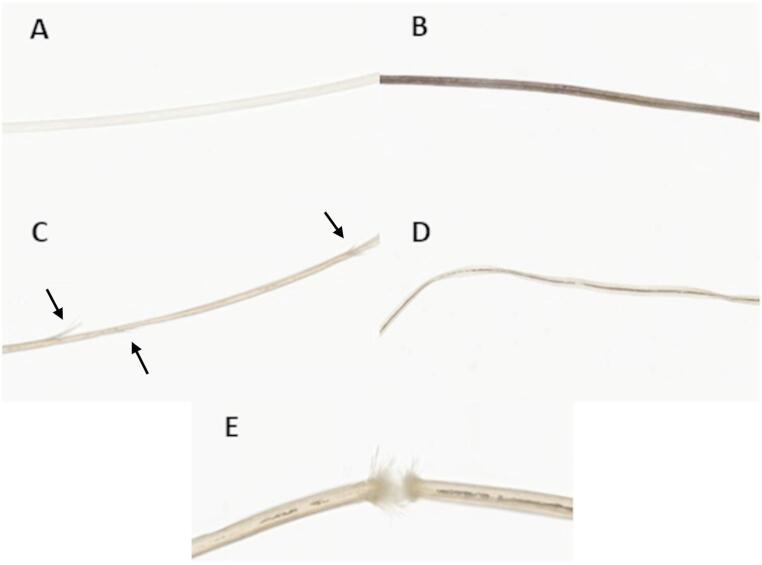
Overview of hair specific characteristics. **A**: Example of a hair with very light pigmentation. **B**: Example of a hair with very dark pigmentation. **C**: Example of a hair with a lot of cuticle irregularities. **D**: Example of a hair with a lot of thickness irregularities. **E**: Example of a hair which displays a breakage

To assess hair specific characteristics, several hairs from the natal cleft were shaved for further microscopic analysis, including thickness, pigmentation, breakage, cuticle irregularities, and hair thickness irregularities (Fig. 2). Thickness and pigmentation was scored on a 5-point scale; 0 meaning very thin/light, respectively, 1 meaning a combination of very thin/average thickness and light hairs/average pigmentation, 2 meaning average thickness or pigmentation, 3 meaning a combination of average thickness/very thick hairs and average pigmentation/dark pigmentation, and 4 meaning very thick/dark. Breakage, cuticle irregularities and hair thickness irregularities were scored on a 3-point scale (0 = no, 1 = a few, 2 = a lot). Microscopic hair analysis was conducted by a dermatopathologist (MD) who was blinded for the origin of the hairs (case vs. control).

#### Statistical analysis

Data were collected and analysed using SPSS for Windows version 29.0 (SPSS Inc., Chicago, IL, USA). The distribution of baseline variables was assessed by using the Shapiro-Wilk test. For variables with a normal distribution, the mean and standard deviation were reported, while for those data not normally distributed, the median and interquartile range (IQR) were presented. For statistical analysis of dichotomous and categorical values between both groups, the Pearson Chi square test was used. To compare continuous data between groups, either an independent samples T-test or a Mann-Whitney U test was conducted, depending on the normality of data distribution.

Univariable analysis was performed on each potential etiologic factor for developing SPSD by calculating odds ratios with 95% confidence intervals. A multivariable logistic regression analysis was conducted including all variables with a *p*-value < 0.20 in univariable analysis. Etiologic factors with a *p*-value < 0.10 in multivariable analysis were considered statistically significant and therefore independent etiologic factors for developing SPSD.

## Results

A total of 100 patients were enrolled in the RCT, 50 patients in each treatment group [9]. For the current study, twelve women included in the RCT were excluded for the current analysis, one patient refused to participate in the trial after inclusion and randomization, and four patients finally did not receive an intervention in the RCT (and therefore were not intra-operatively assessed for the anatomical aspects at the natal cleft). Consequently, a total of 83 male patients with SPSD were available in the case group for the current study. These patients were matched with 83 male controls. SPSD-related symptoms were not observed in any of the individuals in the control group. The median age of the case and control group was 26 (IQR 21–32) and 33 (IQR 28–38), respectively. Since shaved hairs from the natal cleft were only available in 58 cases, hair analysis was only conducted on this subgroup of cases. For hair analysis, these 58 cases were matched with 58 individuals from the control group.

### Potential etiologic factors

Potential etiologic factors for developing SPSD are shown for both groups in Table 1. In the case group, significantly more smokers were present (*n* = 27 vs. *n* = 11, *p* = 0.009). However, the number of cigarettes smoked among the smokers did not significantly differ between the two groups. Twenty patients in the case group (26.7%) had a family history of SPSD, while no one had a family history of SPSD in the control group (*p* < 0.001). BMI and working in sitting position did not significantly differ between both groups.

**Table 1:** Potential etiologic factors for SPSD

	Cases (*n* = 83)	Controls (*n* = 83)	*P*-value
Body Mass Index (kg/m2)	24.2 (IQR 22.6–26.1)	24.4 (IQR 22.5–26.6)	0.946
Smoking (%)	27 (34.2)	11 (14.1)	0.009
No. of cigarettes/day	7.4 (± 6.6)	8.7 (± 7.4)	0.700
Family history of SPSD (%)	20 (26.7)	0 (0.0)	< 0.001
Working sitting position (%)	61 (85.9)	59 (77.6)	0.196
Anatomical aspects of natal cleft			
Depth	3.1 (± 1.1)	3.9 (± 1.1)	< 0.001
Width	1.1 (IQR 0.5–1.6)	0.8 (IQR 0.2–1.3)	0.004
Number of hairs/cm^2^	16 (IQR 11–21)	9 (IQR 6–12)	< 0.001
Microscopic hair aspects ^*^	**(*n*= 58)**	**(*n*= 58)**	
Thickness	2.0 (IQR 1.0–2.0)	1.0 (IQR 1.0–2.0)	0.003
Pigmentation	2.0 (IQR 1.0–2.0)	2.0 (IQR 1.0–2.0)	0.388
Breakage	0.5 (IQR 0.0–1.0)	0.5 (IQR 0.0–1.0)	0.538
Cuticle irregularities	1.0 (IQR 1.0–2.0)	1.0 (IQR 0.0–1.0)	0.025
Thickness irregularities	2.0 (IQR 1.0–2.0)	1.0 (IQR 0.0–1.3)	< 0.001

Values are reported as mean (± standard deviation) or median (interquartile range), unless otherwise stated

*Abbreviations*: *SPSD* sacrococcygeal pilonidal sinus disease, *IQR* interquartile range

^*^Since shaved hairs from the natal cleft were only available in 58 cases, microscopic hair analysis was conducted in 58 cases and 58 matched controls

With regard to the anatomical aspects at the natal cleft, cases had a statistically significant shallower and wider natal cleft (Table 1). Additionally, more hairs were found per square centimeter in the case group (16 vs. 9, *p* < 0.001). Microscopic analysis of the aspects of hairs at the natal cleft revealed that cases had significantly thicker hairs, more cuticle irregularities and more hair thickness irregularities. There were no significant differences with regard to breakage and pigmentation of the hairs at the natal cleft between both groups.

### Uni- and multivariable analysis of potential etiologic factors of SPSD

In univariable analysis, smoking, working in a sitting position, depth and width of the natal cleft, number of hairs at the natal cleft, hair thickness, cuticle irregularities, and hair thickness irregularities were identified as significant potential etiologic factors for developing SPSD (Table 2). BMI, hair pigmentation and hair breakage were not significantly associated with SPSD in univariable analysis. Since a family history of SPSD was observed only in the case group and not at all in the control group, it was not possible to calculate odds ratios, as the extreme odds would have resulted unreliable estimates.

**Table 2:** Uni- and multivariable analysis of etiologic factors for developing sacrococcygeal pilonidal sinus disease

	Univariable logistic regression				Multivariable logistic regression		
	OR		(95% CI)	*p*-value	Adjusted OR	(95% CI)	*p*-value
Body Mass Index (kg/m^2^)	1.0	(−0.08, 0.09)		0.950			
Smoking (yes/no)	3.1	(0.35–1.93)		0.005*	1.6	(−1.19–2.07)	0.598
Working in a sitting position (yes/no)	1.8	(−0.30–1.42)		0.198*	8.8	(0.16–4.18)	0.034*
Anatomical aspects at the natal cleft							
Depth (cm)	0.5	(−0.93 – −0.30)		< 0.001*	0.1	(−3.07 - −0.93)	< 0.001*
Width (cm)	1.5	(0.07–0.79)		0.020*	0.5	(−1.50–0.25)	0.162
Number of hairs/cm^2^	1.2	(0.15–0.30)		< 0.001*	1.4	(0.20–0.54)	< 0.001*
Microscopic aspects at the natal cleft							
Thickness	2.2	(0.25–1.37)		0.005*	2.1	(−0.48–1.98)	0.229
Pigmentation	1.2	(−0.19–0.58)		0.319			
Breakage	1.3	(−0.28–0.74)		0.369			
Cuticle irregularities	1.9	(0.07–1.16)		0.027*	1.8	(−0.50–1.63)	0.298
Hair thickness irregularities	2.5	(0.38–1.45)		< 0.001*	2.1	(−0.37–1.87)	0.191

*Abbreviations:* *OR* odds ratio, *CI* confidence interval

* Significance; *p* < 0.20 in univariable (those variables were included in multivariate analysis) and *p* < 0.10 in multivariable analysis

The potential etiologic factors for having SPSD found to be significant in univariable analysis were all together analysed in multivariable analysis and working in a sitting position, natal cleft depth and the number of hairs at the natal cleft were identified as independent etiologic factors for having SPSD (Table 2).

## Discussion

This study evaluated several potential etiologic factors for the presence of SPSD by comparing patients with SPSD with a control group without SPSD. Smokers and a family history of SPSD were significantly more often present in patients with SPSD compared to controls without SPSD. In addition, the patients with SPSD had a statistically significant shallower and wider natal cleft, significantly more hair at the natal cleft and these hairs were significantly thicker and had significantly more cuticle and thickness irregularities. However, multivariable analysis showed that only working in a sitting position, a shallower natal cleft and more hair at the natal cleft were independently associated with SPSD.

Extensive research has been conducted to unravel the exact etiology of SPSD. While some etiologic factors have been identified and accepted as contributors to SPSD, others remain a subject of debate. Karydakis described three main factors influencing the development of SPSD: loose hair, a force facilitating the penetration of hair, and skin vulnerability [10]. In patients who are hypertrichotic, an increased number of loose hairs can accumulate in the natal cleft, thereby elevating the risk of SPSD development. This aligns with our findings, in which hypertrichotic patients were significantly associated with SPSD. Similar results have been reported in other studies [11, 12]. However, Bosche et al. [13] reported that 74% of hairs found within pilonidal sinus nests are rootless with sharp, cut ends, suggesting that they may originate from the occipital region rather than the natal cleft. A review by Huurman et al. further emphasized the importance of sharp, cut, rootless hairs in the development of SPSD [14]. The natal cleft hair might not directly cause SPSD but may instead play a contributory role. Migrating occipital hairs (i.e., sharp, cut, rootless hairs) may accumulate more readily in patients with a hairier natal cleft than in those with little or no natal cleft hair.

To date, only two other studies have truly evaluated the anatomical characteristics of the natal cleft in patients with SPSD [15, 16]. Akinci et al. observed that patients with SPSD had a deeper natal cleft than patients without SPSD (27 vs. 21 mm), however, Maak et al. concluded that intergluteal fold depth was not associated with an increased risk for developing SPSD. It is important to note that the anatomical measurements were taken in a slightly different way and position compared to the current study. Contrary to their findings, our results indicated that a shallower natal cleft was significantly associated with SPSD. Additionally, the natal cleft was also found to be significantly wider in patients with SPSD compared to the control group in the current study. Following multivariable analysis, regarding the natal cleft anatomy, only a shallower natal cleft was independently associated with SPSD. These findings contradict our initial hypothesis, in which we posited that a more funnel-shaped (deep and narrow) natal cleft would increase the risk for SPSD due to the likelihood of easier hair accumulation in this area, thereby increasing the risk of skin penetration and the formation of a pilonidal sinus. In addition, gluteal flattening procedures, like the Bascom cleft lift procedure, have shown to effectively treat patients with SPSD with low recurrence rates [17]. This also contradicts the finding in the current study that a shallower natal cleft was significantly associated with SPSD. However, the associations of a shallower natal cleft in patients with SPSD compared to the controls observed in our study may be explained by the fact that patients with a shallower natal cleft might experience increased friction from external factors, such as clothing. Moreover, the buttocks may act as a protective factor against microtrauma caused by prolonged sitting in a deeper natal cleft, a protective effect that is less pronounced in patients with a shallower natal cleft. Therefore, the skin might be more vulnerable and prone to facilitating hair penetration when having a shallow cleft [10].

Smoking and BMI are two factors that contribute to the development and persistence of various conditions and diseases due to their systemic effects. Regarding the association with SPSD, only smoking was found to be significantly associated with SPSD in univariable analysis. However, after adjusting for other variables in multivariable analysis, smoking was found not to be an independent etiologic factor in SPSD, likely due to the confounding effects of the other variables. Smoking is a known risk factor for recurrence and complications following pilonidal sinus surgery due to its negative effects on wound healing [18], although not confirmed by all studies [19]. With regard to the development of SPSD, it was previously found that smoking contributes to decreased microvascularization and increased damage to hair follicles, leading to microinflammation [20]. According to the theory as proposed by Bascom, microinflammation of the hair follicles is a contributing factor in the development of SPSD [21]. In line with this, our study reported a significant association between smoking and SPSD. However, this was, as mentioned, only the case in univariable analysis. Obesity has been previously identified as a potential risk factor for SPSD in several studies [6, 22–24]. A possible explanation can also be found in Bascom’s theory, which suggests that enlarged hair follicles result from stretching caused by the gravitational pull on the buttocks due to excess weight [21]. As a person’s weight increases, greater gravitational force is exerted, potentially enlarging hair follicles and thereby increasing the risk of intruding hairs.

Thicker hairs were identified as an etiologic factor for SPSD in univariable analysis. However, when adjusting for other variables in multivariable analysis, hair thickness was not found to be an independent etiologic factor. Nevertheless, the thickness or stiffness of hairs may still contribute to the development of SPSD, particularly in the context of a rising incidence among patients with earlier onset of puberty [25]. Hormonal changes during puberty are known to increase hair thickness and stiffness [26], and an earlier onset of puberty is often associated with higher BMI [25]. Therefore, while hair thickness alone may not independently predict SPSD, it likely acts in combination with other factors, such as BMI, hair density, and anatomical characteristics of the natal cleft, to influence susceptibility to the disease.

This study has some limitations. First, recall bias must be considered in the assessment of a familial history of SPSD. Individuals with SPSD are more likely to discuss their condition with relatives compared to controls, who do not have the condition. As a result, it might be possible that controls are less aware of the occurrence of SPSD within their family. Second, ethnicity was not included in our analysis as a potential etiologic factor for SPSD. Future studies are warranted to investigate this potential etiologic factor. Third, some degree of observer bias is unavoidable during the anatomical measurements of the natal cleft, as the observer could not be blinded to the case or control status of participants. However, since the measurements were conducted in a standardized manner, observer bias was minimized. Lastly, microscopic assessment of the hairs was only conducted by one observer.

## Conclusion

This study has demonstrated that a shallower natal cleft, more hair at the natal cleft and working in a sitting position were independent etiologic factors of SPSD. These findings provide further insights into the etiology of SPSD, which should be considered polyetiological as the etiology is multifaceted and involves many contributing factors. A deeper understanding of the etiology could lead to improved treatment and prevention strategies for SPSD. While not all risk factors identified in this study, such as the shape of the natal cleft, are modifiable, some preventive measures could still be recommended. Keeping the natal cleft free of hair and avoiding prolonged sitting may be advised, particularly for individuals with a known family history of SPSD. Additionally, these etiologic factors might also be valuable in counseling patients with SPSD who opt for non-surgical treatment due to minimal or absence of symptoms.

## Data Availability

No datasets were generated or analysed during the current study.

## Declarations

### Presentation

This manuscript has not been presented elsewhere prior to the submission process.

### Clinical trial registration

This study was not registered as a clinical trial.
